# Inflammatory Index as a Predictor of Mortality in Elderly Patients With Intracapsular Femoral Neck Fracture

**DOI:** 10.7759/cureus.46318

**Published:** 2023-10-01

**Authors:** Aytek Celiksoz, Mustafa Kavak, Ali Okan Tarlacık

**Affiliations:** 1 Orthopedics and Traumatology, Eskişehir City Hospital, Eskişehir, TUR; 2 Orthopedics and Traumatology, Eskişehir Osmangazi University, Eskişehir, TUR

**Keywords:** elderly population, immune-inflammatory index, mortality, hemiarthroplasty, femoral neck fracture

## Abstract

A femoral neck fracture is a major cause of mortality in the elderly population, and intracapsular femoral neck fractures (ICFNFs) are commonly treated with hemiarthroplasty. The 30-day mortality rate for elderly hip fracture patients ranges from 1.0% to 6.5%, and one-year mortality increases significantly to 37.3%. Identifying predictors of mortality in these patients is crucial for better management. Inflammatory indices, such as neutrophil-to-lymphocyte ratio (NLR), systemic immune-inflammation index (SII), and monocyte-to-lymphocyte ratio (MLR), have gained popularity for assessing mortality risk in various diseases. Several studies have demonstrated the value of these indices in predicting mortality after hip fracture. The pan-inflammatory immune value (PIV), which combines hematological parameters, has been shown to predict mortality in cancer patients. However, its role in predicting mortality in ICFNF patients treated with hemiarthroplasty has yet to be explored. This study aimed to assess the association of PIV, SII, NLR, and MLR with 30-day and one-year mortality in ICFNF patients. We also investigated the impact of surgical delay time (≤24h, 24-48h, ≥48h) on these inflammatory indices and mortality. Data from 522 patients with ICFNF treated with hemiarthroplasty were retrospectively collected. We observed 30-day and one-year mortality rates of 5.24% and 21.2%, respectively. Age, gender, and American Society of Anesthesiologists (ASA) score were identified as significant predictors of mortality. Preoperative PIV, SII, NLR, and MLR were significant predictors in the evaluation of early mortality. However, postoperatively, only NLR on the third day (NLR3rd) demonstrated statistical significance. Stepwise logistic regression further confirmed NLR3rd as the most effective predictor for early mortality. For mortality occurring between 30 to 365 days, NLR3rd remained statistically significant, albeit with diminished sensitivity. No other inflammatory index demonstrated significant predictive power for mortality during this later period. Our findings suggest different inflammatory indices may have varying predictive abilities depending on the mortality period. We recommend considering NLR3rd as a valuable and reliable predictor for early mortality in ICFNF patients treated with hemiarthroplasty. Respiratory system disease and preoperative chronic obstructive pulmonary disease (COPD) were identified as risk factors for mortality in our study, in line with previous research. In conclusion, our study highlights the potential of specific inflammatory indexes, particularly NLR3rd, in predicting mortality in elderly patients with ICFNFs treated with hemiarthroplasty. Further research is needed to validate these findings and optimize risk assessment in orthopedic practice.

## Introduction

Femoral neck fractures are a leading cause of death among the elderly population. Intracapsular femoral neck fractures (ICFNFs), in particular, are predominantly treated with hemiarthroplasty. The 30-day mortality rate due to hip fractures in the elderly is reported to range between 1.0% and 6.5%, escalating to 37% at one year [1-3]. Thus, identifying predictors of 30-day and 1-year mortality is crucial in managing these patients. Recent research has focused on developing risk scores that incorporate hematological parameters and inflammation biomarkers, given the pivotal role of inflammation in a myriad of diseases, including atherosclerosis, cardiovascular diseases, chronic heart failure, cancer, and metabolic disorders. Indices such as the neutrophil-to-lymphocyte ratio (NLR), the systemic immune-inflammation index (SII), and the monocyte-to-lymphocyte ratio (MLR) have recently found applications in orthopedic practice. Several studies have demonstrated their value in predicting mortality following hip fractures [4-9]. The pan-immune inflammation value (PIV), defined by Fucà G et al. [10], includes neutrophil, lymphocyte, monocyte, and platelet values and has been shown to be a strong, reliable predictor for colorectal cancer. Several studies have demonstrated the utility of PIV as a prognostic tool in conditions such as acute myocardial infarction [11], breast cancer, Merkel cell carcinoma, and metastatic renal cell carcinoma [12]. Thus, PIV holds the potential as a reliable predictor of risk for elderly patients undergoing hemiarthroplasty due to a femoral neck fracture. To the best of our knowledge, no prior research has investigated the efficacy of PIV in predicting mortality in cases of ICFNF treated with hemiarthroplasty. The main aim of this study was to determine the association of PIV, SII, NLR, and MLR with 30-day mortality and one-year mortality in elderly patients with ICFNF. The effect of a delay to surgery of ≤24h, 24-48h, or ≥48 h on the inflammatory indexes and mortality rates was also investigated. 

## Materials and methods

Study design

**Figure 1:**
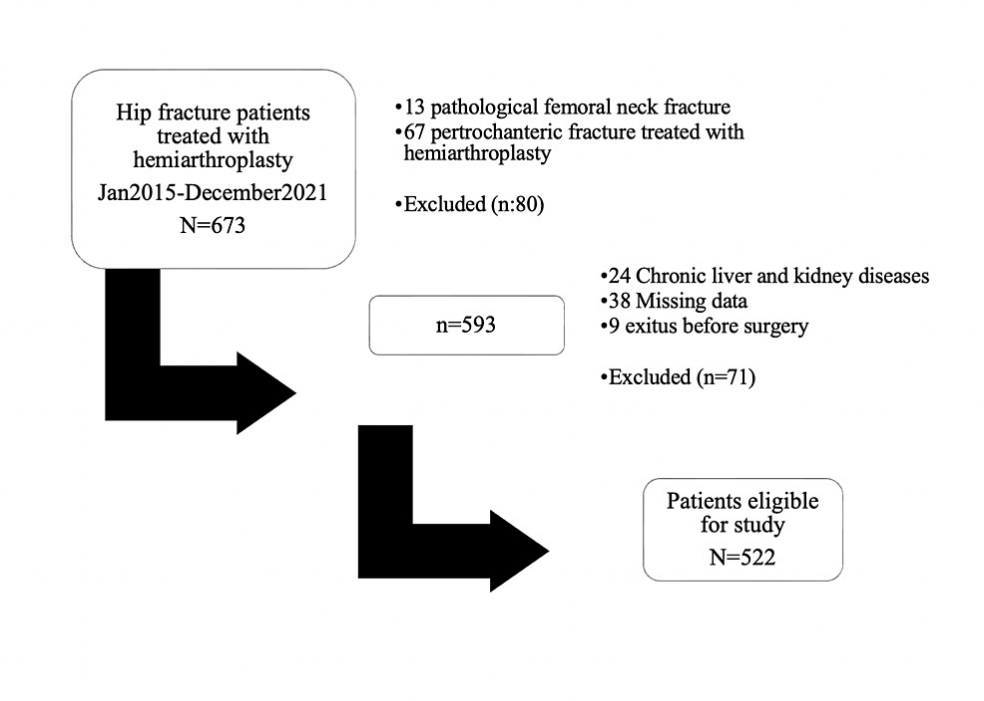
Patient selection process.

We conducted a retrospective analysis of data from patients who presented with an ICFNF at the tertiary hospital between January 2015 and December 2021. The study included patients aged 65 years or above who experienced an ICFNF due to a fall from height or other low-energy trauma, presented within one week of the fracture, and had a follow-up period of at least one year. The exclusion criteria were subtrochanteric or intertrochanteric fractures, intraoperative fractures, ipsilateral lower extremity fractures, pathological fractures, high-energy trauma, cancer, chronic liver disease, renal failure, or myocardial infarction within the past year. A total of 673 patients with ICFNF were initially recorded. After the exclusion of 151 patients, the study was conducted with the remaining 522 patients' data. All patients were allowed to mobilize with full weight-bearing on the first postoperative day. Postoperatively, routine antibiotic prophylaxis was administered as cefazolin 3x1 gr IV, and low molecular weight heparin was given as 2x4000 IU subcutaneously for 35 days. Patients' clinical characteristics and demographic data, including ASA scores and comorbid disease consultations, were recorded. We assessed the patients for 30-day and one-year mortality. A delay to surgery was also evaluated in three groups of ≤24 hrs, 24-48 hrs, and ≥48 hrs (Figure 1). 

Laboratory findings

Upon admission to the ED, blood tests were routinely conducted for all patients with femoral neck fractures. Postoperative blood samples were collected on the first and third days of hospitalization. The parameters recorded included platelet count (x10^9/L, reference range 100-300), hemoglobin (g/L, reference range 130.0-175.0), lymphocyte count (x10^9/L, reference range 1.1-3.2), albumin (g/L, reference range 35.0-55.0), neutrophil count (x10^9/L, reference range 42.3-77.8), monocyte count (x10^9/L, reference range 0.4-1.2), creatinine (μmol/L, reference range 53.0-106.0), and C-reactive protein (mg/L). Calculations were conducted for PIV: [Neutrophil count x Platelet count x Monocyte count] / Lymphocyte count [10], SII: [Neutrophil count x Platelet count] / Lymphocyte count [6], NLR: Neutrophil count / Lymphocyte count [13], and MLR: Monocyte count / Lymphocyte count [14]. We compared the PIV, SII, NLR, and MLR values of patients with 30-day mortality to those with follow-ups after 30 days and compared these indices between patients with 1-year mortality to those with follow-ups after one year. The preoperative and postoperative changes in PIV, SII, NLR, and MLR were analyzed in relation to the time to surgery (≤24 hrs, 24-48 hrs, ≥ 48 hrs). These changes were referred to as 'delta' PIV (DPIV) and 'delta' SII (DSII), respectively. Patients were monitored for a minimum of one-year post-admission, and any occurrences of 30-day or one-year all-cause mortality were recorded from the hospital database. In-hospital deaths were categorized as 30-day mortality. All relevant data were then compared between the different groups. The study evaluated 522 patients with ICFNF who underwent hemiarthroplasty and met the study criteria.

Statistical analysis

Statistical analyses were conducted using SPSS Statistics version 22.0 software (IBM Corp., Armonk, NY, USA). Descriptive statistics for numerical variables are presented as mean, standard deviation (SD), and range (minimum-maximum values). Given that the values did not exhibit normal distribution, as determined by the Shapiro-Wilk test for normality, non-parametric test procedures were employed. The Mann-Whitney U test was utilized for comparisons, and the Kruskal-Wallis variance analysis was employed to discern relationships between the parameters. Receiver operating characteristic (ROC) curve analysis was used to determine the cut-off values. The effect of the presence of comorbid disease on mortality was evaluated with binary logistic regression analysis. The results were evaluated within a 95% CI, and a p-value <0.05 was considered statistically significant.

## Results

The mortality rates were 5.74% (n:30) at 30 days and 21.26% (n:111) at one year. No statistically significant differences were observed between the groups with 30-day mortality, one-year mortality, and survivors concerning the type of anesthesia and delay to surgery (p=0.76, p=0.31, respectively). However, statistically significant differences were found in terms of age, gender, and ASA score (p≤0.001, p=0.02, p≤0.001, respectively). The average age of patients who experienced 30-day mortality was 83.20 years, with a statistically significant cut-off value determined to be 86.0 years (p≤0.001). The average age of patients who experienced one-year mortality was 80.41 years, with the statistically significant cut-off value determined to be 72.0 years (p≤0.001). According to the results of logistic regression analysis, being aged ≥86 years increased the risk of 30-day mortality by 2.817 times. Gender was identified as a risk factor for both 30-day and one-year mortality in patients with ICFNF treated with hemiarthroplasty. The statistical analysis results showed a significant association between gender and mortality (p=0.02). A higher mortality rate was determined in males than in females. In the statistical analysis of this study, an ASA score of 3 was found to be a significant predictor of increased risk for 30-day and one-year mortality (p≤0.01, p≤0.01, respectively). When examining the impacts of comorbid diseases on 30-day and one-year mortality, the presence of cardiac diseases, confirmed with cardiology consultation, and pulmonary diseases such as COPD and pneumonia, confirmed with pulmonary consultation, showed statistical significance (p=0.03, p=0.02; p=0.01, p=0.01 respectively). However, other comorbid diseases and the associated consultations (including nephrology, neurology, endocrinology, and gastroenterology) did not exhibit statistical significance (Table 1).

**Table 1:** Demographic data (Chi-Square test). ASA: American Society of Anesthesiologists.

	30-day mortality (n:30)	One-year mortality (n:111)	Survivor (n:381)	Total (n:522)	P-value (Monte Carlo)
Age	83.20 ± 6.83	80.41 ± 9.24	77.48 ±9.00	78.43 ± 9.08	≤0.001
Gender					0.02
Male	12 (40%)	51 (45.9%)	113 (29.7%)	176	
Female	18 (60%)	60 (54.1%)	267 (70.1%)	345	
Anesthesia					0.76
General	2 (6.7%)	8 (7.2%)	33 (8.7 %)	43 (8.2%)	
Spinal	15 (50.0%)	68 (61.3%)	220 (57.7%)	303 (58.0%)	
Epidural	13 (43.3%)	35 (31.5%)	128 (33.6%)	176 (33.7%)	
*ASA					≤0.001
1	0	0	17 (4.5%)	17 (3.3%)	
2	15 (50.0%)	77 (69.4%)	314 (82.4 %)	406 (77.8%)	
3	14 (46.7%)	32 (28.8%)	95 (18.2%)	95 (18.2%)	
4	1 (0.3%)	1 (0.3%)	2 (1.8%)	4 (0.8%)	
Delay to surgery					0.31
0-24 hours	11 (36.7%)	41 (36.9%)	173 (45.4%)	225 (43.1%)	
24-48 hours	4 (13.3%)	25 (22.5%)	66 (17.3%)	95 (18.2%)	
≥48 hours	15 (50.0%)	45 (40.5%)	142 (37.3%)	202 (38.7%)	

In the 30-day mortality group, the variables of PIV^preop^, SII^preop^, NLR^preop^, NLR^3rd^, and MLR^preop^ were determined to be statistically significant (p=0.02, p= 0.01, p=0.00, p=0.00, p=0.00, respectively). The ROC analysis calculated the cut-off values as 777.2, 1014.86, 4.75, 8.49, and 0.73, respectively (Table 2).

**Table 2:** The 30-day mortality using ROC curve analysis. ROC: Receiver operating characteristic curve; PIV^Preop^: Preoperative pan immune-inflammation value; PIV^1st^: Pan immune-inflammation value postoperative 1st day; PIV^3rd^: Pan immune-inflammation value postoperative 3rd day; SII^Preop^: Preoperative systemic immune-inflammation index; SII^1st^: Systemic immune-inflammation index postoperative 1st day; SII^3rd^: Systemic immune-inflammation index postoperative 3rd day; NLR^Preop^: Neutrophil-lymphocyte ratio preoperative; NLR^1st^: Neutrophil-lymphocyte ratio postoperative 1st day; NLR^3rd^: Neutrophil-lymphocyte ratio postoperative 3rd day.

	Cut-off	AUC	Sensitivity (%)	Specificity (%)	P-value
Age	86	0.613	34.04	84.51	≤0.001
PIV^ Preop^	772.2	0.564	65.25	47.63	0.023
PIV^1st^	1578.23	0.503	47.14	57.41	0.908
PIV^3rd^	745.78	0.517	75.47	32.5	0.618
SII^ Preop^	1014.86	0.569	77.3	36.48	0.012
SII^1st^	2341.5	0.519	45.0	63.49	0.514
SII^3rd^	2071.0	0.549	45.28	67.92	0.147
NLR^ Preop^	4.75	0.579	78.01	37.27	0.003
NLR^1st^	11.44	0.537	41.43	67.72	0.197
NLR^3rd^	8.49	0.607	57.94	61.25	0.001
MLR^ Preop^	0.73	0.576	39.72	75.79	0.007
MLR^1st^	0.82	0.523	50.71	59.52	0.424
MLR^3rd^	1.22	0.507	86.92	20.0	0.845

In the one-year mortality group, NLR3rd was determined to be statistically significant (p=0.048), and the cut-off value was calculated as 6.49 with a sensitivity of 80% in the ROC analysis (Table 3).

**Table 3:** The 30-365 day mortality (ROC curve analysis). ROC: Receiver operating characteristic curve; PIV^Preop^: Preoperative pan immune-inflammation value; PIV^1st^: Pan immune-inflammation value postoperative 1st day; PIV^3rd^: Pan immune-inflammation value postoperative 3rd day; SII^Preop^: Preoperative systemic immune-inflammation index; SII^1st^: Systemic immune-inflammation index postoperative 1st day; SII^3rd^: Systemic immune-inflammation index postoperative 3rd day; NLR^Preop^: Neutrophil-lymphocyte ratio preoperative; NLR^1st^: Neutrophil-lymphocyte ratio postoperative 1st day; NLR^3rd^: Neutrophil-lymphocyte ratio postoperative 3rd day.

	Cut-off	AUC	Sensitivity (%)	Specificity (%)	P-value
Age	72.0	0.655	96.67	28.66	≤0.001
PIV^ Preop^	3696.0	0.563	16.67	94.5	0.784
PIV^1st^	494.85	0.563	24.14	88.14	0.272
PIV^3rd^	327.97	0.538	25.0	90.18	0.623
SII^ Preop^	1014.86	0.535	80.0	33.54	0.546
SII^1st^	2741.14	0.506	37.93	71.17	0.909
SII^3rd^	1092.0	0.502	85.0	26.07	0.971
NLR^ Preop^	14.34	0.530	26.67	86.38	0.602
NLR^1st^	10.41	0.545	55.17	60.53	0.377
NLR^3rd^	6.69	0.609	80.0	42.51	0.048
MLR^ Preop^	0.41	0.531	53.33	65.17	0.612
MLR^1st^	0.5	0.582	44.83	75.87	0.174
MLR^3rd^	0.35	0.519	30.0	87.77	0.799

**Figure 2:**
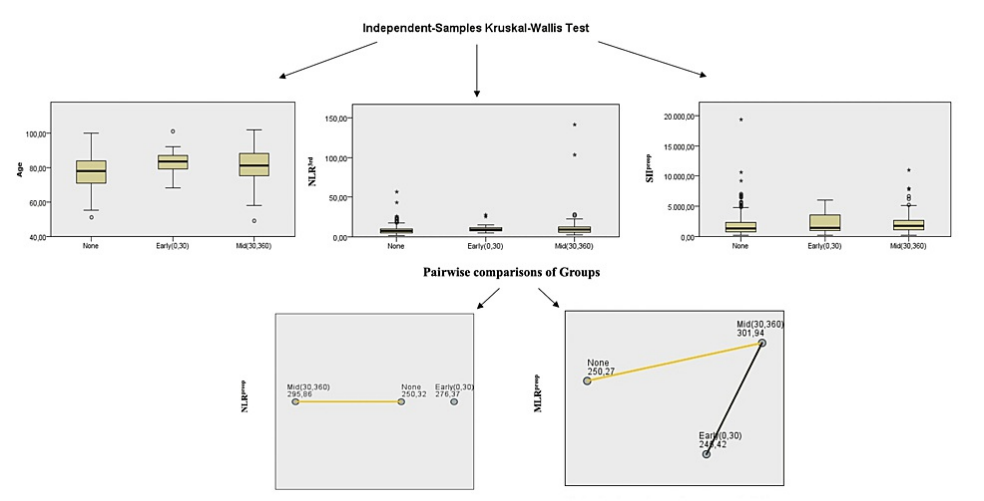
Comparison of three groups using the independent sample Kruskal-Wallis test. The Kruskal-Wallis test was employed to assess the statistical significance of several variables across the groups, including: age, SII^preop^, NLR^preop^, NLR^3rd^, and MLR^preop^. NLR^3rd^: Neutrophil-lymphocyte ratio postoperative 3rd day; SII^Preop^: Preoperative systemic immune-inflammation index; NLR^Preop^: Neutrophil-lymphocyte ratio preoperative.

In this study, comparisons were made between three groups based on 30-day mortality (Group 1), one-year mortality (Group 2), and survivors (Group 3). The Kruskal-Wallis test was employed to assess the statistical significance of several variables across the groups, including age, SIIpreop, NLRpreop, NLR3rd, and MLRpreop (Table 4, Figure 2). Multiple comparisons were made for each group: Age: Group 1 and Group 2 (p=0.00); SIIpreop: Group 2 and Group 3 (p= 0.01); NLRpreop: Group 2 and Group 3 (p=0.01); NLR3rd: Group 2 and Group 3 (p=0.00); MLRpreop: Group 2 and Group 3 (p=0.00).

**Table 4:** Evaluation of the effects of inflammation indexes on 30-day, 1-year mortality, and survivors (pairwise multiple comparison). *p <0.05 denotes statistical significance. **Multiple comparison: Groups compared pairwise. PIV^Preop^: Preoperative pan immune-inflammation value; PIV^1st^: Pan immune-inflammation value postoperative 1st day; PIV^3rd^: Pan immune-inflammation value postoperative 3rd day; SII^Preop^: Preoperative systemic immune-inflammation index; SII^1st^: Systemic immune-inflammation index postoperative 1st day; SII^3rd^: Systemic immune-inflammation index postoperative 3rd day; NLR^Preop^: Neutrophil-lymphocyte ratio preoperative; NLR^1st^: Neutrophil-lymphocyte ratio postoperative 1st day; NLR^3rd^: Neutrophil-lymphocyte ratio postoperative 3rd day.

	30-day Mortality (Group 1)	30-365 day Mortality (Group 2)	Survivors (Group 3)		
	Median (Min/Max)	Median (Min/Max)	Median (Min-Max)	P*	Multiple Comparison**
Age	83.20 ± 6.83	80.41 ± 9.24	77.48 ±9.00	0.06	Group1-2 (P=0.00)
PIV^ Preop^	520.4 (20.71-5376.6)	616.2 (56.07-9957.9)	446.3 (27.84-17442)	0.064	
PIV^1st^	541.0 (24.57-6102.42)	807.9 (147.5-21229.7)	707.9 (48.96-36500)	0.434	
PIV^3rd^	386.9 (45.73-8340.55)	784.3 (168.75-8140.2)	585.5 (65.11-9887.3)	0.638	
SII^ Preop^	1019.4 (230.12-5974)	1032 (193.25-11000)	763.1 (180.27-19380)	0.048	Group 2-3 (P=0.01)
SII^1st^	1174.5 (557.7-6780.4)	1163.1 (295-15164)	1152.6 (163.20-18684.0)	0.795	
SII^3rd^	1138.5 (457.25-8970)	1161 (120.3-12503.3)	1026.6 (162.7-12183.3)	0.295	
NLR^ Preop^	4.06 (1.41-37)	5.04 (1.76-33.33)	3.53 (1.02-60)	0.017	Group 2-3 (P=0.01)
NLR^1st^	6.4 (3.46-18.86)	6.30 (2.11-49.23)	5.84 (0.80-86.5)	0.415	
NLR^3rd^	6.8 (4.8-27.6)	5.77 (2-141.54)	5.11 (1.02-56.67)	0.006	Group 2-3 (P=0.01)
MLR^ Preop^	0.293 (0.03-2)	0.29 (0.17-3.5)	0.33 (0.07-4.5)	0.005	Group 2-3 (P=0.00)
MLR^1st^	0.348 (0.8-2.83)	0.55 (0.20-5)	0.50 (0.12-5.89)	0.107	
MLR^3rd^	0.312 (0.18-1.68)	0.46 (0.20-5.21)	0.47 (0.10-3.30)	0.950	

In the study, the time elapsed until surgery was categorized into three groups: 0-24 hours, 24-48 hours, and ≥48 hours. No statistically significant relationship was found when comparing early and delayed mortality within these time groups. In further analyses, the changes in indexes between preoperative and postoperative day 1 were denoted as 'delta' (Δ). In the comparisons of surgeries performed within 0-24 hours and ≥48 hours, significant statistical differences were observed in the ΔPIVpreop-1st, ΔSIIpreop-1st, and ΔNLRpreop-1st indexes (p=0.00, p=0.00, and p=0.00, respectively). The ΔMLRpreop-1st value was not statistically significant according to the time to surgery (p=0.45).

In the 30-day mortality group, the stepwise logistic regression results showed that among the inflammatory indices, NLR3rd was found to be statistically significant in the final step. This suggests that NLR3rd is a significant predictor of mortality within the first 30 days (p=0.01) (Table 5). The analysis of one-year mortality showed that no particular inflammatory index exhibited superiority over the others (Table 6). 

**Table 5:** 30-day mortality with stepwise logistic regression analysis. 'a' represents a cascading process to identify the most significant index. Variables entered in step 1 include: Preop PIV, PAN1st, PAN3rd, Preop SII, SII1st, SII3rd, Preop NLR, NLR1st, NLR3rd, Preop MLR, MLR1st, MLR3rd, and Delay to surgery. The last step, denoted as 'a', reveals the most significant index. PIV^Preop^: Preoperative pan immune-inflammation value; PIV^1st^: Pan immune-inflammation value postoperative 1st day; PIV^3rd^: Pan immune-inflammation value postoperative 3rd day; SII^Preop^: Preoperative systemic immune-inflammation index; SII^1st^: Systemic immune-inflammation index postoperative 1st day; SII^3rd^: Systemic immune-inflammation index postoperative 3rd day; NLR^Preop^: Neutrophil-lymphocyte ratio preoperative; NLR^1st^: Neutrophil-lymphocyte ratio postoperative 1st day; NLR^3rd^: Neutrophil-lymphocyte ratio postoperative 3rd day.

Variables in the Equation								
		B	S.E. of B	Wald Statistics	P-value	Exp (p)	95% CI for Exp (p)	
							Lower	Upper
First Step^a^	PIV^preop^	0.000	0.000	0.010	0.919	1.000	1.000	1.000
	PIV^1st^	0.000	0.000	0.274	0.601	1.000	1.000	1.000
	PIV^3rd^	0.000	0.000	0.960	0.327	1.000	1.000	1.001
	SII^preop^	0.000	0.000	0.001	0.975	1.000	0.999	1.000
	SII^1st^	0.000	0.000	0.012	0.911	1.000	1.000	1.000
	SII^3rd^	0.000	.000	1.397	0.237	1.000	0.999	1.000
	Preop NLR	0.010	.053	.035	0.851	1.010	0.911	1.119
	NLR^1st^	-0.007	0.047	0.021	0.884	0.993	0.906	1.089
	NLR^3rd^	0.134	0.059	5.072	0.024	1.143	1.018	1.285
	Preop MLR	0.181	0.448	0.163	0.687	1.198	0.498	2.883
	MLR^1st^	0.434	0.400	1.179	0.278	1.544	0.705	3.382
	MLR^3rd^	-1.249	0.530	5.548	0.019	0.287	0.101	0.811
	Delay to surgery	0.002	0.003	0.730	0.393	1.002	0.997	1.008
	Constant	-1.308	0.327	16.018	0.000	0.270		
Last Step^a^	NLR^3rd^	0.048	0.019	6.042	0.014	1.049	1.010	1.090
	Constant	-1.269	0.215	34.842	0.000	0.281		

**Table 6:** Mortality between 30 and 365 days postoperative assessed with stepwise logistic regression analysis. 'a' cascading to find the most significant index: Variable(s) entered on step 1: PIV^Preop^, PAN^1st^, PAN^3rd^, SII^Preop^, SII^1st^, SII^3rd^, NLR^Preop^, NLR^1st^, NLR^3rd^, MLR^Preop^, MLR^1st^, MLR^3rd^, delay to surgery. Last step 14 'a': There was no most significant index. *PIV^Preop^: Preoperative pan immune-inflammation value; PIV^1st^: Pan immune-inflammation value postoperative 1st day; PIV^3rd^: Pan immune-inflammation value postoperative 3rd day; SII^Preop^: Preoperative systemic immune-inflammation index; SII^1st^: Systemic immune-inflammation index postoperative 1st day; SII^3rd^: Systemic immune-inflammation index postoperative 3rd day; NLR^Preop^: Neutrophil-lymphocyte ratio preoperative; NLR^1st^: Neutrophil-lymphocyte ratio postoperative 1st day; NLR^3rd^: Neutrophil-lymphocyte ratio postoperative 3rd day.

		B	S.E.	Wald	Sig.	Exp (B)	95% CI for Exp (B)	
							Lower	Upper
Step 1^a^	PIV^Preop^*	0.000	0.001	0.120	0.729	1.000	0.999	1.001
	PIV^1st^	-0.001	0.000	1.502	0.220	0.999	0.999	1.000
	PIV^3rd^	0.001	0.000	2.832	0.092	1.001	1.000	1.001
	Preop SII	0.000	0.001	0.158	0.691	1.000	0.998	1.001
	SIIS^1st^	0.000	0.001	0.000	0.988	1.000	0.999	1.001
	SIIS^3rd^	0.000	0.000	0.539	0.463	1.000	0.999	1.000
	Preop NLR	0.123	.114	1.156	0.282	1.130	0.904	1.413
	NLR^1st^	0.031	.090	0.122	0.727	1.032	0.865	1.230
	NLR^3rd^	0.035	.038	0.840	0.359	1.035	0.961	1.116
	Preop MLR	-1.488	1.400	1.130	0.288	0.226	0.015	3.510
	MLR^1st^	0.098	1.106	0.008	0.929	1.103	0.126	9.645
	MLR^3rd^	-0.992	0.922	1.158	0.282	0.371	0.061	2.258
	Delay to surgery	0.005	0.005	0.841	0.359	1.005	0.995	1.015
	Constant	-2.499	0.698	12.818	0.000	0.082		
Step 14^a^	Constant	-2.791	0.230	146.806	0.000	0.061		

## Discussion

The key findings of this current study demonstrated differences in the inflammation indexes for predicting 30-day and one-year mortality in elderly patients with ICFNF. According to the evaluations, preoperative PIV, SII, NLR, and MLR were found to be statistically significant predictors of early mortality (0-30 days). However, postoperatively, only NLR^3rd^ demonstrated statistical significance. The significance of NLR^3rd^ exclusively among the indexes suggests that the postoperative bleeding and inflammation processes following ICFNFs, together with the effects of arthroplasty, may have distinct impacts on platelet levels and inflammatory markers. The consistent finding of NLR^3rd^ being the only index with statistical significance is further supported by the stepwise logistic regression analysis results, highlighting NLR^3rd^ as a more effective predictive marker than the other indexes. Consequently, it can be concluded that NLR^3rd^ can be a valuable and reliable predictive factor for early mortality in orthopedic practice, particularly for patients with ICFNF. This insight may significantly contribute to clinical decision-making and risk assessment in orthopedic care. Kolhe SN et al. showed that admission NLR was not a predictive factor for early mortality. In contrast, the current study results showed that both NLR^preop^ and NLR^3rd^ were good predictors for early mortality. However, the current study was limited to the specific fracture type of ICFNF [15]. In a meta-analysis by Chen YH et al., NLR was not found to be significant for postoperative mortality [16]. In the current study's evaluations of late mortality (one-year mortality), only NLR^3rd^ of the inflammatory indexes demonstrated statistical significance, with a sensitivity of 80%. However, when evaluated using stepwise logistic regression, no inflammatory index was statistically significant in predicting late mortality. This suggests that while NLR^3rd^ has some predictive power for late mortality, it might not be a strong predictor for this particular period. Furthermore, no other inflammatory index was found to be statistically significant in predicting late mortality. These results indicate that different inflammatory indexes may have varying predictive abilities depending on the specific mortality period being considered. The findings of studies by Bingol O et al. and Temiz A and Ersözlü S showed differences in the significance of NLR as a predictor for one-year mortality. However, the results in the current study appear quite different. To establish NLR as an independent risk factor for one-year survival in geriatric ICFNFs, it is essential to account for other factors that may influence mortality. To ensure the validity and reliability of the PIV, SII, NLR, and MLR as prognostic indexes, controlling for confounding variables could impact mortality outcomes [8,17].

Giannoulis D et al. reported a 30-day mortality rate in the range of 1.4%-10%, whereas the rate in the current study was 5.74%. Johnson-Lynn S et al. reported a one-year mortality of 30.7%, which, in the current study, was 21.26% [18,19]. Age of ≥86 years and male gender were considered serious risk factors for 30-day mortality in patients with ICFNF treated with hemiarthroplasty in the current study. In a study of a Scottish population, Holt G et al. also reported male gender as a risk factor for mortality after hip fracture, similar to the current study population [20]. Barceló M et al. reported the most common causes of death to be respiratory system and secondary circulatory system failure two years after hip fracture. Pneumonia has been reported to be a risk factor for mortality after elderly hip fracture surgery, and COPD has been reported to be an independent risk factor for one-year mortality after hip fracture surgery [21-23]. The current study showed that respiratory system disease was at risk for mortality at all times of death (upper limit 95% CI: 0.015, p=0.01), and preoperative COPD was also a risk factor for ICFNF treated with hemiarthroplasty (upper limit 95% CI: 0.00, p=0.00). Similarly, Barceló M et al. stated that cardiology system disease was statistically significant for mortality (upper limit 95% CI: 0.036, p=0.03). Several studies have reported and demonstrated that early surgery in hip fractures reduces mortality rates [24-27]. Our current study evaluated the delay to surgery time by dividing it into three groups (≤24, 24-48, ≥48 hours). Contrary to previous studies, no statistically significant difference was observed between early and late mortality according to the time of surgery.

This intriguing finding prompted a more detailed investigation, and the changes in inflammatory indexes were examined in each group. To achieve this, the change was denoted as 'delta' (Δ). Notably, when comparing surgeries performed within 0-24 hours and ≥48 hours, statistically significant differences were observed in the ΔPIV^preop-1st^, ΔSII^preop-1st^, and ΔNLR^preop-1st^ indexes (p=0.00, p=0.00, and p=0.00, respectively). However, ΔMLR^preop-1st^ did not show statistically significant differences based on the time of surgery (p=0.45). These strong and statistically significant changes in inflammatory indexes indicate the profound impact of early surgery on the systemic inflammatory and immune systems. This observation supports the notion that early surgery might significantly affect the overall physiological response. Further investigations are warranted to elucidate the underlying mechanisms responsible for the differences observed in the inflammatory indexes and their potential associations with early surgery. Understanding these mechanisms could lead to more effective strategies for managing hip fractures and improving patient outcomes.

Strengths and limitations 

This study had the limitation of being a retrospective observational study with a relatively small sample size. However, a strong aspect was that it is the first study to have used PIV in orthopedic femoral neck fractures and compared four inflammatory indexes. It is also the first to have demonstrated the significance of inflammatory changes 'Δ' in relation to the time to surgery.

## Conclusions

This study highlights the increased mortality risk in patients aged 86 years and older with ICFNF. PIV shows promise as a novel marker for early mortality prediction in orthopedic practice, although NLR remains more effective. It is important to emphasize that this study has laid the foundation for further extensive research in the context of predicting one-year mortality. Establishing inflammatory biomarkers as independent prognostic factors requires a deeper exploration of their intricate roles and dynamics within the complex interplay of physiological and pathological processes. Future studies should address the long-term prognostic value of different inflammatory markers in a larger and more diverse patient population to determine their true potential in predicting longer-term outcomes, such as one-year mortality. In particular, pulmonary disease, primarily COPD, significantly increases the risk in hip fracture patients, highlighting the need for integrated pulmonary and orthopedic care. Understanding these factors is essential to refine prognostic models and improve outcomes in orthopedic practice.
